# Primary pelvic soft tissue sarcomas (PELVISARC): outcomes from the TransAtlantic Australasian Retroperitoneal Sarcoma Working Group (TARPSWG)

**DOI:** 10.1093/bjs/znae128

**Published:** 2024-05-28

**Authors:** Marco Fiore, Catherine Sarre-Lazcano, Myles Smith, Misbah Khan, Chandrajit P Raut, Charles Honoré, Paul Sargos, Ferdinando Cananzi, Giovanni Grignani, Shintaro Iwata, Alessandro Gronchi, TransAtlantic Australasian Retroperitoneal Sarcoma Working Group, Max Almond, Dan Blazer, Sylvie Bonvalot, Antoine Bouchard-Fortier, Dario Callegaro, Ferdinando Cananzi, Kenneth Cardona, Chiara Colombo, Mark Fairweather, Matthieu Faron, Marco Fiore, Dorian Yarih Garcia-Ortega, Giovanni Grignani, Alessandro Gronchi, David Gyorki, Charles Honoré, Shintaro Iwata, Misbah Khan, Gary Mann, Audrey Michot, Carolyn Nessim, Marko Novak, Vittorio Quagliuolo, Sergio Damian Quildrian, Stefano Radaelli, Chandrajit Raut, Piotr Rutkowski, Laura Samà, Paul Sargos, Catherine Sarre-Lazcano, Jacek Skoczylas, Myles Smith, Dirk Strauss, Mario Terlizzi, Dimitri Tzanis, Sergio Valeri, Winan J van Houdt, Jiping Wang, Michelle Wilkinson

**Affiliations:** Department of Surgery, Fondazione IRCCS Istituto Nazionale dei Tumori, Milan, Italy; Department of Surgery, Instituto Nacional de Ciencias Médicas y Nutrición Salvador Zubirán General Surgery, Mexico City, Mexico; Sarcoma Unit, Royal Marsden Hospital, London, UK; Sarcoma Unit, Royal Marsden Hospital, London, UK; Dana-Farber Cancer Institute, Brigham and Women’s Hospital, Harvard Medical School, Boston, Massachusetts, USA; Department of Surgical Oncology, Institut Gustave Roussy, Paris, France; Department of Radiotherapy, Institut Bergonié, Bordeaux, France; Sarcoma, Melanoma and Rare Tumors Surgery Unit, IRCCS Humanitas Research Hospital, Milan, Italy; Department of Biomedical Sciences, Humanitas University, Milan, Italy; Division of Medical Oncology, Candiolo Cancer Institute, Candiolo, Italy; Department of Musculoskeletal Oncology and Rehabilitation, National Cancer Center Hospital, Tokyo, Japan; Department of Surgery, Fondazione IRCCS Istituto Nazionale dei Tumori, Milan, Italy

## Introduction

Adult pelvic soft tissue sarcomas (PSTS) have often been included among retroperitoneal sarcomas (RPS) in previously published series to date. PSTS comprise 0.3% of all sarcomas and 5–18% of abdominal and RPS cases^1,2^. Recently, collaborative multi-institutional groups have published information about tumour biology, natural history, prognosis and management recommendations^3,4^. A proposed operative definition of adult PSTS includes those arising from non-visceral pelvic structures, delineated anteriorly by the pubis and inguinal ligament, posteriorly by the sacrum, superiorly by the parietal peritoneum and inferiorly by the pelvic floor^5^. This anatomical region exhibits constraints and complex relationships with surrounding organs and structures that potentially can influence disease progression, treatment and prognosis^6^.

Histologic type, grade, optimal surgical treatment, margin status and treatment at a high-volume referral centre have been shown to influence prognosis in STS^7,8^. The site of origin also impacts on prognosis^2^, thus prompting a dedicated analysis of PSTS. Whether recent recommendations for RPS are applicable to PSTS needs evaluation. The primary aim of this study is to outline the patterns of presentation, treatment modalities and long-term outcomes of PSTS cases treated at TransAtlantic Australasian Retroperitoneal Sarcoma Working Group (TARPSWG) centres. Secondary aims of the study include description of the extent of surgery and postoperative outcomes together with analysis of the prognostic factors unique to PSTS.

## Methods

A retrospective analysis of primary, non-metastatic PSTS in patients over 18 years old treated between 2005 and 2018 at 22 TARPSWG centres was performed. PSTS were defined as tumours originating from non-visceral pelvic structures, with possible extension into the abdominal cavity and herniation through the sciatic notch, the obturator foramen and/or inguinal ligament. Gastrointestinal stromal tumours (GISTs), gynaecological, urogenital, Ewing family sarcomas and desmoids were excluded. Patients presenting with metastatic disease less than 3 months after the initial surgery were considered as metastatic at diagnosis and therefore excluded, as well as those with initial surgical treatment outside of a TARPSWG centre.

Patients’ demographics, clinical, histological, surgical and treatment characteristics were collected. Extent of resection was classified as macroscopically complete (R0/R1) or incomplete (R2). Resection of adjacent organs, specifying musculoskeletal, nervous, visceral or vascular structures, was recorded. Surgical complications were graded according to the modified Clavien–Dindo classification^9^. Tumour size, histologic type and grade according to the Federation Nationale des Centres de Lutte Contre le Cancer (FNCLCC) were obtained from the surgical specimen, or by imaging studies and initial biopsy in non-surgical cases. According to the World Health Organization criteria^10^, histologic types were grouped as follows: well-differentiated liposarcoma (WDLPS), dedifferentiated liposarcoma (DDLPS), leiomyosarcoma (LMS), solitary fibrous tumour (SFT) and others. Occurrence, timing and type of perioperative treatment with chemotherapy (CT) or radiotherapy (RT) was obtained. Patient follow-up was updated until September 2021.

The Kruskal–Wallis test was used to compare numerical variables. Pearson’s chi-squared test was used to compare categorical variables in the surgical cohort, whereas Fisher’s exact test was used for the non-surgical cohort. Local recurrence-free survival (LRFS), distant metastasis-free survival (DMFS), overall survival (OS) and disease-specific survival (DSS) were calculated, and survival analysis was performed using the Kaplan–Meier method and log rank test. Uni- and multivariable regression analyses were performed to identify prognostic factors. Statistical significance was set as *P* < 0.05. The statistical analysis excluded patients with missing data and was performed using SPSS software version 26. Pertinent Internal Review Board approval for this study was obtained at participating centres.

## Results

### Patients’ characteristics

Overall, 448 patients were identified, with a median of 11 patients per centre (interquartile range [IQR] 6–42). Surgical resection was performed in 401 (89.5%) patients, in whom 349 (87%) had a preoperative biopsy. The operated patients constituted the cohort of interest for the primary analysis of this study. Median age was 57 years (IQR 46–67), 252 (62.8%) were male and 357 (89%) had an Eastern Cooperative Oncology Group (ECOG) performance status < 2. Clinical and surgical characteristics classified by histologic type are shown in *Table 1*. Leiomyosarcoma was the most frequent histologic type (24%), with 28 (28.6%) arising from a named vein, followed by solitary fibrous tumour (18%). WDLPS and DDLPS accounted for 15% and 17% of cases respectively. Other histological types included 22 (5.4%) malignant peripheral nerve sheath tumours (MPNST), 18 (4.4%) undifferentiated pleomorphic sarcoma (UPS), 13 (3.2%) spindle cell sarcoma, 12 (2.9%) myxofibrosarcoma, 11 (2.7%) myxoid liposarcoma and 7.4% corresponding to other infrequent histotypes.

**Table 1:** Clinical and surgical variables

	Surgical patients *n* = 401	WDLPS *n* = 59	DDLPS *n* = 67	LMS *n* = 98	SFT *n* = 71	Others *n* = 106	*P*
Sex							<0.001
Male	252 (62.7)	36 (61.0)	49 (73.1)	47 (48.0)	56 (78.9)	64 (60.4)	
Female	149 (37.3)	23 (39.0)	18 (26.9)	51 (52.0)	15 (21.1)	42 (39.6)	
Age (years), median (IQR)	57 (46–67)	62 (52–70)	61 (50–69)	60 (52–67)	52 (45–60)	52 (36–66)	<0.001
ECOG							0.061
0	224 (55.9)	37 (62.7)	32 (47.8)	57 (58.2)	51 (71.8)	47 (44.3)	
1	133 (33.2)	19 (32.2)	26 (38.8)	31 (31.6)	15 (21.1)	42 (39.6)	
2	38 (9.5)	2 (3.4)	8 (11.9)	8 (8.2)	5 (7.0)	15 (14.2)	
3	4 (1.9)	0	1 (1.5)	1 (1.0)	0	2 (1.9)	
Tumour size (mm), median (IQR)	130 (80–180)	195 (150–230)	160 (110–210)	100 (64–150)	110 (70–160)	113 (80–173)	<0.001
Laterality							<0.001
Left	154 (38.4)	24 (40.7)	34 (50.7)	41 (41.8)	17 (23.9)	38 (35.8)	
Right	144 (35.9)	28 (47.5)	20 (29.9)	36 (36.7)	18 (25.4)	42 (39.6)	
Central	103 (25.7)	7 (11.9)	13 (19.4)	21 (21.4)	36 (50.7)	26 (24.5)	
FNCLCC Grade							<0.001
G1	123 (30.7)	59	0	16 (16.3)	36 (50.7)	14 (13.2)	
G2	140 (34.9)	0	52 (77.6)	39 (39.8)	24 (33.8)	24 (22.6)	
G3	120 (29.9)	0	14 (20.9)	40 (40.8)	2 (2.8)	64 (60.4)	
Resected organs							<0.001
0	59 (14.7)	19 (32.2)	3 (4.5)	5 (5.1)	19 (26.8)	13 (12.3)	
1	80 (20.0)	14 (23.7)	11 (16.4)	14 (14.3)	19 (26.8)	22 (20.8)	
2	73 (18.2)	14 (23.7)	8 (11.9)	21 (21.4)	11 (15.5)	19 (17.9)	
3	74 (18.5)	9 (15.3)	14 (20.9)	26 (26.5)	8 (11.3)	17 (16.0)	
≥4	115 (28.7)	3 (5.1)	31 (46.3)	32 (32.7)	14 (19.7)	35 (33.0)	
Median (IQR)	2 (1–3)	1 (0–2)	3 (2–6)	3 (2–4)	1 (0–3)	2 (1–4)	
Type of resection							
Visceral	215 (53.6)	17 (28.8)	43 (64.2)	68 (69.4)	40 (56.3)	47 (44.3)	<0.001
Urologic	121 (30.2)	6 (10.2)	31 (46.3)	38 (38.8)	22 (31.0)	24 (22.6)	<0.001
Musculoskeletal	193 (48.1)	27 (45.8)	48 (71.6)	38 (38.8)	20 (28.2)	60 (56.6)	<0.001
Vascular	84 (20.9)	7 (11.9)	20 (29.9)	36 (36.7)	6 (8.5)	15 (14.2)	<0.001
Nervous	47 (11.7)	4 (6.8)	12 (17.9)	4 (4.1)	1 (1.4)	26 (24.5)	<0.001
Clavien–Dindo complications ≥3	78 (19.5)	7 (11.9)	18 (26.9)	15 (15.3)	13 (18.3)	25 (23.6)	0.146
ICU admission	92 (22.9)	7 (11.9)	14 (20.9)	28 (28.6)	22 (31)	21 (19.8)	0.055

Values are *n* (%) unless otherwise indicated.

Median operating time was 240 min (IQR 180–360). Resections were macroscopically complete (R0/R1) in 90.8% cases. Intraoperative sarcomatosis was found in 13 (3.2%) patients, being unresectable in 2 cases. Peritoneal invasion was found in 26 cases (6.5%), with neither showing a statistically significant difference between histologic types. Tumour rupture was reported in 35 (8.7%) cases.

**Fig. 1:**
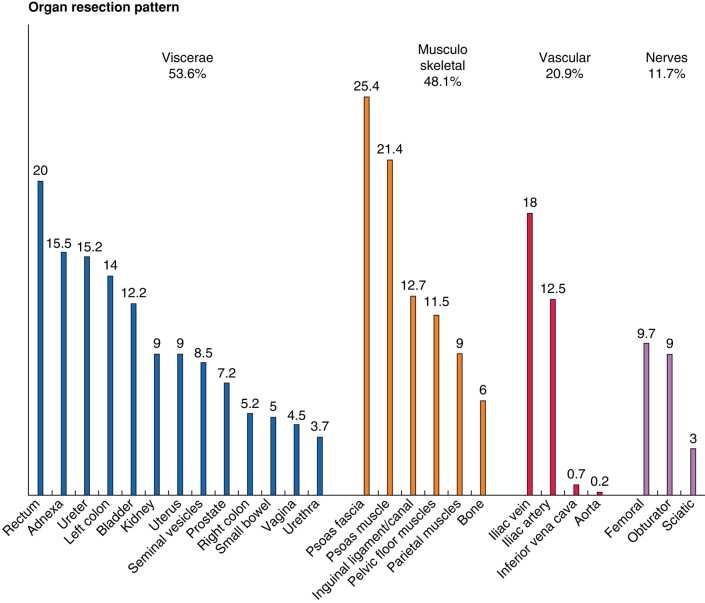
Adjacent organ resection pattern (%)

Adjacent organ resection pattern is shown in *Fig. 1*. Visceral resection was required in 215 (53.6%), musculoskeletal resection in 193 (48.1%), vascular resection in 84 (20.9%) and nerve resection in 47 (11.7%) cases. Total pelvic exenteration was done in 9 (2.2%) cases. This subgroup comprised eight male and one female patient, whose ages ranged from 36 to 69 years. Intestinal stomas were required in 66 (16.4%) patients, an end colostomy in 33 (8.2%) cases, a diverting ileostomy in 18 (4.5%) cases, or a colostomy in 15 (3.7%) cases. A urinary stoma was necessary in 25 (6.2%) cases. Severe complications, defined as Clavien–Dindo grade ≥ 3, occurred in 78 patients (19.5%), with the most common being deep surgical site infection/collection (38 cases, 9.5%), followed by bleeding (33 cases, 8.2%) and sepsis (23 cases, 5.7%). Bowel and urinary anastomotic leaks were reported in 9 (2.2%) and 12 (3%) cases respectively. Deep venous thrombosis was seen in 15 (3.7%) cases, whereas lymphatic leak was reported in only 5 cases (1.2%). Reoperation was needed in 41 (10.2%) cases. Mean hospital stay was 9 days (IQR 7–15) and 30-day mortality rate was 2.7%, with no statistically significant difference among histologic types.

### Multimodal treatment

**Fig. 2:**
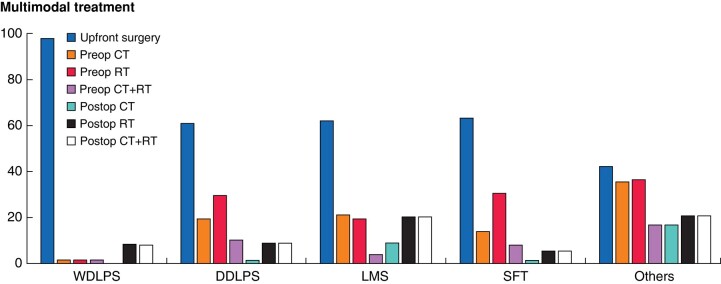
Multimodal treatment pattern (%)

Neoadjuvant or adjuvant therapy was administered in 205 (51.1%) cases, with 104 patients (25.9%) receiving chemotherapy and 162 (40.4%) radiotherapy (RT). Concomitant chemotherapy and radiotherapy was given in 68 (16.9%) patients. Treatment modality, timing and frequency significantly differed (*P* < 0.0001) across histologies, as shown in *Fig. 2*.

Preoperative RT was administered in 101 (25.2%) cases, postoperative RT in 57 (14.2%), preoperative RT + postoperative boost in 3 (0.7%) cases and intraoperative RT + postoperative boost in 1 (0.3%) case. Mean RT dose was 50 Gy (standard deviation [SD] 6.5, range 30–70). The most common reasons for not administering RT were institutional policies in 129 cases (54.0%), physician’s preference in 74 cases (31.0%), potential risk of toxicity in 13 cases (5.4%), patient too symptomatic in 10 cases (4.3%), patient refusal in 5 cases (2.1%) and others (8 cases, 3.3%).

Neoadjuvant chemotherapy was administered in 83 (20.7%) cases. The most common chemotherapy regimen was anthracycline/ifosfamide in 55 cases (13.7%), followed by dacarbazine in 13 cases (3.2%), high-dose ifosfamide in 12 cases (3%), single-agent anthracycline in 11 cases (2.7%) and gemcitabine/docetaxel in 5 cases (1.2%), with 10 patients (2.5%) receiving other regimens.

The most common reasons for not administering neoadjuvant chemotherapy were institutional policy in 219 cases (68.9%), physician’s preference in 72 cases (22.6%), patient too symptomatic in 16 cases (5%), potential risk of toxicity in 2 cases (0.6%) and others (9 cases, 2.8%). Adjuvant chemotherapy was administered in 29 patients (5.2%), with the most frequent regimens being anthracycline/ifosfamide in 17 cases (4.2%), single-agent anthracycline in 7 cases (1.7%) and dacarbazine in 3 cases (0.7%).

Analysis of eight major high-volume centres treating at least 15 patients in the study period found high variability in pattern of multimodal strategies adopted (*Fig. S1*). These institutions were located in Europe (6), North America (1) and Asia (1).

### Follow-up, recurrence pattern and survival

**Fig. 3:**
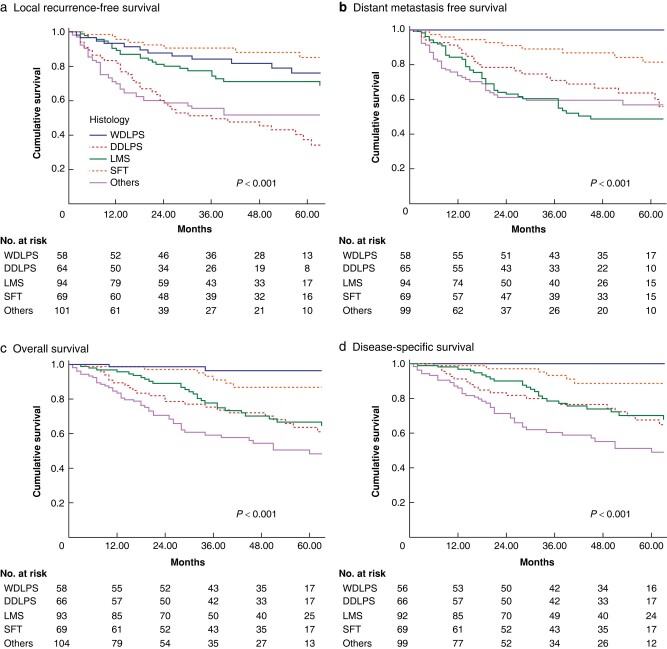
**Five-year LRFS, DMFS, OS and DSS according to histology**. **a** Local recurrence-free survival, **b** distant metastasis-free survival, **c** overall survival and **d** disease-specific survival according to histology

With a median follow-up of 53 months (IQR 30–87), 135 (33.7%) patients developed local recurrence (LR) and 126 (31.4%) distant metastasis (DM). Five-year LRFS was 62.7% (standard error [SE] ±2.8), 5-year DMFS was 66.1% (SE ±2.7), 5-year OS was 69.6% (SE ±2.6) and 5-year DSS was 72.3% (SE ±2.6). Different survival was observed according to histological type (*Fig. 3*).

The most frequent locations for recurrence were local (93 cases, 68.9%), pelvic multifocal (18 cases, 13.3%), sarcomatosis (11 cases, 8.1%) and pelvic contralateral (5 cases, 3.7%), with only three patients (2.2%) presenting retroperitoneal extra-pelvic recurrence. Time to LR was different between histological type (*P* < 0.001), with the shortest survival seen in the other histology group (median 8 months, IQR 4–13), followed by LMS (18, 10–31), DDLPS (18, 8–34), SFT (21, 11–74) and WDLPS (23, 8–52). Early LR (<12 months after surgery) were not attributable to a specific subtype in the other histology group, even though several pleomorphic liposarcomas and MPNST had an early LR.

The most frequent sites of DM were the lung in 68 (53.9%), liver in 23 (18.2%) and distant sarcomatosis in 15 (11.9%) cases. Median time to DM was significantly shorter in the less-frequent histology group (8 months, IQR 4–15, *P* = 0.01), followed by DDLPS (16, 10–38), LMS (18, 9–37) and SFT (25, 8–56). Only one WDLPS patient presented DM at 65 months.

Multivariable regression analysis (*Table 2*) showed significantly higher LR, DM and mortality risk for Grade 3 tumours (HR 6.35, 8.22 and 7.6, *P* < 0.001). DDLPS was the histology at highest risk of LR (HR 4.09, *P* = 0.004) and LMS at highest risk of DM (HR 2.34, *P* = 0.04).

**Table 2:** Univariate and multivariate analysis of competing risk for local recurrence, distant metastasis and disease-specific mortality

	Univariate analysis				Multivariate Analysis			
	HR	95% CI		*P*	HR	95% CI		*P*
**Local recurrence**								
Age	1.01	1.00	1.03	0.10	1.01	0.99	1.03	0.10
Size	1.00	0.99	1.00	0.37	0.99	0.99	1.002	0.58
FNCLCC grade (*versus* 1)								
Grade 2	3.66	2.00	6.70	<0.001	3.81	1.42	10.2	0.008
Grade 3	5.27	2.85	9.76	<0.001	6.35	2.29	17.5	<0.001
Histology (*versus* SFT)								
WDLPS	1.89	0.77	4.66	0.16	3.34	1.03	10.76	0.04
DDLPS	9.03	3.94	20.6	<0.001	4.09	1.58	10.59	0.004
LMS	2.20	0.98	4.92	0.055	0.93	0.36	2.38	0.88
Others	4.5	2.08	9.73	<0.001	2.11	0.83	5.36	0.11
**Distant metastasis**								
Age	1.00	0.98	1.01	0.97	0.99	0.97	1.01	0.55
Size	0.99	0.99	1.00	0.08	0.99	0.99	1.00	0.66
FNCLCC grade (*versus* 1)								
Grade 2	7.48	3.49	16.0	0.001	4.23	1.77	10.08	0.001
Grade 3	14.4	6.71	31.1	0.001	8.22	3.3	20.4	<0.001
Histology (*versus* SFT)								
WDLPS	0.08	0.01	0.67	0.02	0.16	0.19	1.44	0.10
DDLPS	2.74	1.23	6.08	0.01	1.14	0.46	2.85	0.76
LMS	5.12	2.45	10.6	<0.001	2.34	1.02	5.37	0.04
Others	2.86	1.37	5.97	0.005	0.96	0.40	2.31	0.93
**Disease-specific mortality**								
Age	1.01	0.99	1.02	0.21	1.00	0.99	1.02	0.37
Size	0.99	0.99	1.00	0.56	1.00	0.99	1.00	0.77
FNCLCC grade (*versus* 1)								
Grade 2	10.9	4.16	28.6	<0.001	3.83	1.34	10.89	0.01
Grade 3	23.6	8.97	62.0	<0.001	7.6	2.63	21.99	<0.001
Histology (*versus* SFT)								
WDLPS	–	–	–	–	–	–	–	–
DDLPS	6.17	2.45	15.4	<0.001	2.48	0.90	6.82	0.07
LMS	5.18	2.13	12.5	<0.001	2.24	0.84	5.90	0.10
Others	6.29	2.62	15.0	<0.001	2.31	0.85	6.25	0.09
**Overall mortality**								
Age	1.01	0.99	1.02	0.21	1.02	1.00	1.03	0.01
Size	0.99	0.99	1.00	0.56	1.00	0.99	1.00	0.91
FNCLCC grade (*versus* 1)								
Grade 2	10.9	4.16	28.6	<0.001	2.52	1.04	6.09	0.04
Grade 3	23.6	8.9	62.0	<0.001	4.91	1.99	12.11	0.001
Histology (*versus* SFT)								
WDLPS	0.50	0.14	1.71	0.27	0.58	0.14	2.31	0.44
DDLPS	5.58	2.39	13.0	<0.001	2.43	0.94	6.30	0.06
LMS	4.59	2.04	10.3	<0.001	2.08	0.85	5.09	0.10
Others	5.28	2.37	11.7	<0.001	2.29	0.91	5.78	0.07

### Non-surgical patients

In the 47 (10.5%) patients that were not eligible for surgery other modalities of treatment were offered. Tumour characteristics and treatment modalities according to histology are shown in *Table S2* (Supplement). These patients had significantly higher frequency of ECOG ≥2 when compared to the surgical cohort (*P* = 0.0001), with no differences found regarding age, tumour size or grade.

## Discussion

This retrospective analysis of PSTS highlights distinct surgical anatomy, histologic distribution and prognosis, setting it apart from previous RPS literature. Variations in organ resection patterns, coupled with frequent demands for significant vascular and nerve resections, emphasizes the necessity of high-volume, multidisciplinary teams for treatment, as well as the paramount importance of a preoperative biopsy. Previously, PSTS have been included in RPS studies, potentially missing their distinctive differences in presentation, treatment and prognosis due to the influence that anatomic site may have a prognostic impact on local disease control. This multi-institutional series highlights key differences between PSTS and RPS that have been published^11^.

**Fig. 4:**
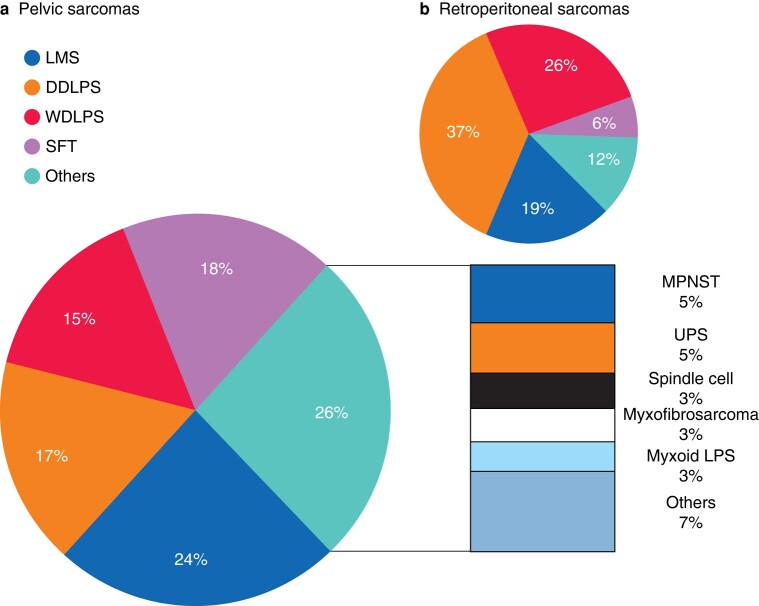
**Histology distribution of PSTS compared to RPS series**. **a** Histology distribution of PSTS. **b** Histology distribution of RPS. From: TARPSWG, *Ann Surg* 2016;263(5):1002–1009. DDLPS = dedifferentiated liposarcoma, LMS = leiomyosarcoma, MPNST = malignant peripheral nerve sheath tumour, SFT = solitary fibrous tumour, UPS = undifferentiated pleomorphic sarcoma, WDLPS = well-differentiated liposarcoma

Histologically, PSTS were more frequently LMS and SFT (24 and 18% respectively), whereas in the retroperitoneum the most common subtypes were DDLPS and WDLPS (37 and 26% respectively)^11^, as depicted in *Fig. 4*. This histologic difference has been noted in similar but smaller series^12^. Different organ resection patterns are also evident between PSTS and RPS. In PSTS, pelvic structures such as the rectum (20.0%), adnexa (15.5%) and ureter (15.2%) were the most frequently resected organs, whereas the kidney (54.8%), left colon/rectum (32.7%) and right colon (24.5%) predominate in multivisceral resections for RPS. Bladder resection rates, including partial bladder resections as well as cystectomy, are notably higher in PSTS than in RPS (12.2% *versus* 1.8%). Vascular involvement similarly differs, with PSTS necessitating iliac vein/inferior vena cava resections more frequently than RPS (18.7% *versus* 10.9%), along with iliac artery/aorta resections (12.7% *versus* 3.2%). Major nerve resections also differ, as PSTS cases more often required femoral, obturator or sciatic nerve resections (11.7%), whereas major lumbar nerve resection was needed in 5–6% of RPS patients^11^.

The complexity of pelvic anatomy, tumour biology and organ infiltration might render the principles of RPS compartmental resection^13^ less applicable to PSTS. Collaboration with vascular^14^, orthopaedic^15^ and plastic surgery specialists is essential when performing extensive primary resections^16^. The role of pelvic imaging review to guide resection extent in cases with unclear structural threat or involvement is yet to be ascertained^17^. The relationship between tumour biology, histology and surgical planning should be considered, as the risk of local recurrence is highest for DDLPS^18^.

Because the update of the consensus on the treatment of RPS has shifted its focus towards modulating treatment approach by histological type, rather than conducting a specific analysis of anatomical sites such as the pelvis, it is necessary to find new collaboration strategies in order to achieve advancements in the approach to pelvic sarcomas. A promising strategy might involve fostering increased collaboration between the sarcoma expert community and the ongoing collaborative efforts in complex pelvic surgery for advanced gynaecological and rectal cancers^19^, to improve patient outcome. In this context, it is crucial to acknowledge that the necessity for pelvic exenteration emerged in a subset of merely nine patients within this series, representing 2.2% of cases. Consequently, the imperative for collaborative efforts extends beyond mere expertise in the established and codified exenteration procedure. It potentially involves a broader exploration of safer and more nuanced approaches to resection, encompassing the intricate structures of the pelvic floor.

In accordance with recent consensus guidelines and recommendations^4,20^, the majority of patients underwent a preoperative biopsy. This facilitated inclusion of the histologic type as well as the proximity to critical neurovascular and musculoskeletal structures, in decisions about neoadjuvant therapy use that may improve the rate of R0/R1 resections^21,22^. However, the proportion of R2 resection in the present series (9.2%) is almost two-fold higher than that reported from the most recent data on pooled RPS series^11^. Examining multimodal therapy, the use of neo- or adjuvant chemotherapy or radiotherapy was more pronounced in PSTS (25.9% and 40.4% respectively) compared to the combined RPS-PSTS series (18.2% and 32%). This distinction is likely attributable to the histological differences observed. Multimodal therapy also differed according to histology, with higher rates being used in ‘other’ PSTS subtypes, followed by LMS. Multimodal therapy warrants consideration within multidisciplinary case discussion, particularly for chemosensitive histologies and borderline resectable patients^4^. RT should also be carefully considered, given the higher prevalence of SFT within PSTS^23^.

The data present real-world insight into the approaches at TARPSWG centres, demonstrating a high variability with the use of multimodal therapy. Regrettably, no randomized trials have been undertaken to specifically investigate the multimodal strategy in the context of pelvic sarcoma. Consequently, the data at hand serve as the most pertinent reference point currently available. Should the goal be to enhance the efficacy and precision of preoperative strategies, these findings provide a benchmark for analysis. In fact, pelvic location was not an exclusion criterion in the STRASS trial investigating the role of preoperative RT in RPS. Similarly, PSTS were not excluded from enrolment in the ongoing STRASS2 trial investigating neoadjuvant chemotherapy in high-risk LPS and LMS patients. Nonetheless, no specific analysis focusing on pelvic location was planned in either trial, and no granular details are available.

In the TARPSWG RPS report, an RPS-PSTS series demonstrated a 5-year LRFS of 74.1%, DMFS of 79% and OS of 67%^11^. In this PSTS series, we note similar OS (69.6%), whereas LRFS (62.7%) and DMFS (66.5%) were lower. Although ‘other’ less-frequent histologic types exhibited the shortest time to LR, the pattern of local and distant recurrence aligned with histology, mirroring RPS trends. DDLPS carried the highest LR risk, whereas LMS exhibited the highest risk of DM. Notably, the WDLPS subgroup experienced no sarcoma-related deaths, distinguishing it from purely retroperitoneal WDLPS.

This study bears limitations due to its retrospective nature and logistical challenges in harmonizing data across international centres. Despite this, ongoing collaboration among these institutions facilitated a large data set. Prospective patient registries will be instrumental in validating observed PSTS differences and standardizing treatment and surgical strategies. The RESAR project (NCT03838718), led by TARPSWG, will offer high-quality prospective data on RPS and PSTS surgical approaches^24^ in the coming years. Further research areas include quality-of-life measurements, as well as patient-related outcomes, including recurrent and non-operative patients.

The findings of this study imply that the inherent progression trajectory and surgical intervention strategies pertaining to PSTS warrants distinct consideration from those applied to RPS cases. Moreover, the inclusion of anatomical location, alongside histologic type and tumour biology, should be integral when formulating future clinical trials to facilitate better analysis of this subgroup, and thereby inform the best treatment strategies.

## Collaborators

Max Almond0000-0003-4133-312X, Dan Blazer III0000-0002-7261-9254, Sylvie Bonvalot0000-0001-9726-9732, Antoine Bouchard-Fortier0000-0002-8920-5429, Dario Callegaro0000-0002-3392-4002, Ferdinando Cananzi0000-0002-8227-3373, Kenneth Cardona0000-0002-8200-3269, Chiara Colombo0000-0001-8031-5528, Mark Fairweather0000-0003-4705-6645, Matthieu Faron0000-0003-2331-0792, Marco Fiore0000-0001-8220-424X, Dorian Yarih Garcia-Ortega0000-0002-1302-2896, Giovanni Grignani0000-0001-5515-569X, Alessandro Gronchi0000-0002-4703-3534, David Gyorki0000-0002-3165-4694, Charles Honoré0000-0003-1184-6143, Shintaro Iwata0000-0002-2024-514X, Misbah Khan0000-0001-6076-8415, Gary Mann0000-0003-3874-3267, Audrey Michot0000-0001-6486-0091, Carolyn Nessim0000-0002-0419-3511, Marko Novak0000-0002-1524-1478, Vittorio Quagliuolo0000-0002-6102-0000, Sergio Damian Quildrian0000-0001-5794-7084, Stefano Radaelli0000-0001-8181-3641, Chandrajit Raut0000-0001-7297-3221, Piotr Rutkowski0000-0001-8009-0816, Laura Samà0000-0001-9036-7444, Paul Sargos0000-0002-7301-0870, Catherine Sarre-Lazcano0000-0002-0048-6659, Jacek Skoczylas0000-0002-8920-5429, Myles Smith0000-0002-3254-4817, Dirk Strauss0000-0003-2413-0697, Mario Terlizzi0000-0001-8496-6918, Dimitri Tzanis0000-0001-8090-1391, Sergio Valeri0000-0002-2589-3904, Winan J. van Houdt0000-0002-2303-3468, Jiping Wang0000-0002-7466-098X, Michelle Wilkinson0000-0001-8155-2883

M. Almond (University Hospitals Birmingham, Birmingham, UK); D. Blazer III (Duke Cancer Center, Durham, NC, USA); S. Bonvalot (Institut Curie, Paris, France); A. Bouchard-Fortier (Alberta Health Services, University of Calgary, Calgary, AL, Canada); D. Callegaro (Fondazione IRCCS Istituto Nazionale dei Tumori, Milan, Italy); F. Cananzi (IRCCS Humanitas Research Hospital, Humanitas University, Rozzano, Italy); K. Cardona (Emory University, Atlanta, GE, USA); C. Colombo (Fondazione IRCCS Istituto Nazionale dei Tumori, Milan, Italy); M. Fairweather (Brigham and Women's Hospital, Dana-Farber Cancer Institute, Harvard Medical School, Boston, MA, USA); M. Faron (Institut Gustave Roussy, Villejuif, France); M. Fiore (Fondazione IRCCS Istituto Nazionale dei Tumori, Milan, Italy); D.Y. Garcia-Ortega (National Cancer Institute, Mexico City, Mexico); Giovanni Grignani (AOU CIttà della Salute e della Scienza di Torino, Turin, Italy); A. Gronchi (Fondazione IRCCS Istituto Nazionale dei Tumori, Milan, Italy); D. Gyorki (Peter MacCallum Cancer Center, Melbourne, Australia); C. Honoré (Institut Gustave Roussy, Villejuif, France); S. Iwata (National Cancer Center Hospital, Tokyo, Japan); M. Khan (Royal Marsden Hospital, London, UK); G. Mann (Roswell Park Comprehensive Cancer Center, Buffalo, NY, USA); A. Michot (Institut Bergonié, Bordeaux, France); C. Nessim (The Ottawa Hospital, OHRI, University of Ottawa, Ottawa, ON, Canada); M. Novak (Institute of Oncology Ljubljana, Ljubliana, Slovenia); V. Quagliuolo (IRCCS Humanitas Research Hospital, Rozzano, Italy); S.D. Quildrian (University of Buenos Aires - Angel H Roffo Institute of Oncology/ Buenos Aires British Hospital, Buenos Aires, Argentina); C. Raut (Brigham and Women's Hospital, Dana-Farber Cancer Institute, Harvard Medical School, Boston, MA, USA); S. Radaelli (Fondazione IRCCS Istituto Nazionale dei Tumori, Milan, Italy); P. Rutkowski (Maria Sklodowska-Curie National Research Institute of Oncology, Warsaw, Poland); L. Samà (IRCCS Humanitas Research Hospital, Rozzano, Italy); P. Sargos (Institut Bergonié, Bordeaux, France); C. Sarre-Lazcano (Instituto Nacional de Ciencias Médicas y Nutrición Salvador Zubirán, Mexico City, Mexico); J. Skoczylas (Maria Sklodowska-Curie National Research Institute of Oncology, Warsaw, Poland); M. Smith (Royal Marsden Hospital, London, UK); D. Strauss (Royal Marsden Hospital, London, UK); M. Terlizzi (Institut Bergonié, Bordeaux, France); D. Tzanis (Institut Curie, Paris, France); S. Valeri (Fondazione Policlinico Universitario Campus Bio-Medico, Rome, Italy); W.J. van Houdt (Nederland Cancer Institute, Amsterdam, The Netherlands); J. Wang (Brigham and Women's Hospital, Dana-Farber Cancer Institute, Harvard Medical School, Boston, MA, USA); M. Wilkinson (Peter MacCallum Cancer Center, Melbourne, Australia)

## Supplementary Material

znae128_Supplementary_Data

## Data Availability

Complete data are available upon justified request to the Corresponding Author. Results of this study have been communicated as a poster during Connective Tissue Oncology Society Annual Meeting in Vancouver, BC, Canada, 16–19 November 2022
