# Supplementary material for: Primary pelvic soft tissue sarcomas (PELVISARC): outcomes from the TransAtlantic Australasian Retroperitoneal Sarcoma Working Group (TARPSWG)
Source: Br J Surg. 2024 May 28;111(5):znae128. doi: 10.1093/bjs/znae128 (PMC11132121; doi:10.1093/bjs/znae128)
Supplement: znae128_Supplementary_Data [file znae128_supplementary_data.docx]

**PRIMARY PELVIC SOFT TISSUE SARCOMAS (PELVISARC): OUTCOMES FROM THE TRANSATLANTIC AUSTRALASIAN RETROPERITONEAL SARCOMA WORKING GROUP (TARPSWG)**

Marco Fiore^1^, Catherine Sarre-Lazcano^2^, Myles Smith^3^, Misbah Khan^3^, Chandrajit P. Raut^4^, Charles Honore^5^, Paul Sargos^6^, Ferdinando Cananzi^7,8^, Giovanni Grignani^9^, Shintaro Iwata^10^, Alessandro Gronchi^1^ on behalf of TransAtlantic Australasian Retroperitoneal Sarcoma Working Group.

^1^ Fondazione IRCCS Istituto Nazionale dei Tumori, Department of Surgery, Milan, Italy
^2^ Instituto Nacional de Ciencias Médicas y Nutrición Salvador Zubirán General Surgery, Mexico City, Mexico
^3^ Royal Marsden Hospital, Sarcoma Unit, London, United Kingdom
^4^ Brigham and Women’s Hospital, Dana-Farber Cancer Institute, Harvard Medical School, Boston, Massachusetts, USA
^5^ Department of Surgical Oncology, Institut Gustave Roussy, Paris, France
^6^ Department of Radiotherapy, Institut Bergonié, Bordeaux, France
^7^ Sarcoma, Melanoma and Rare Tumors Surgery Unit, IRCCS Humanitas Research Hospital, Rozzano, Milan, Italy
^8^ Department of Biomedical Sciences, Humanitas University, Pieve Emanuele, Milan, Italy.
^9^ Candiolo Cancer Institute, Division of Medical Oncology, Candiolo, Italy
^10^ National Cancer Center Hospital, Department of Musculoskeletal Oncology and Rehabilitation, Tokyo, Japan

**Corresponding author.** Marco Fiore, MD, FACS, FEBSh. Sarcoma Service, Fondazione IRCCS Istituto Nazionale dei Tumori. Via Venezian, 1 – I-20133 Milan, Italy

**ORCID ID:** 0000-0001-8220-424X

**Twitter**: @FioreDoc

**Supplementary Materials - Index**

| **Supplementary Figures and Tables** |  |
| --- | --- |
| Figure S1 | *page 2* |
| Table S2 | *page 3* |
|  |  |

**Supplementary Figures and Tables**

**Figure S1.** Variability in RT and CT use in high-volume TARPSWG centers

**
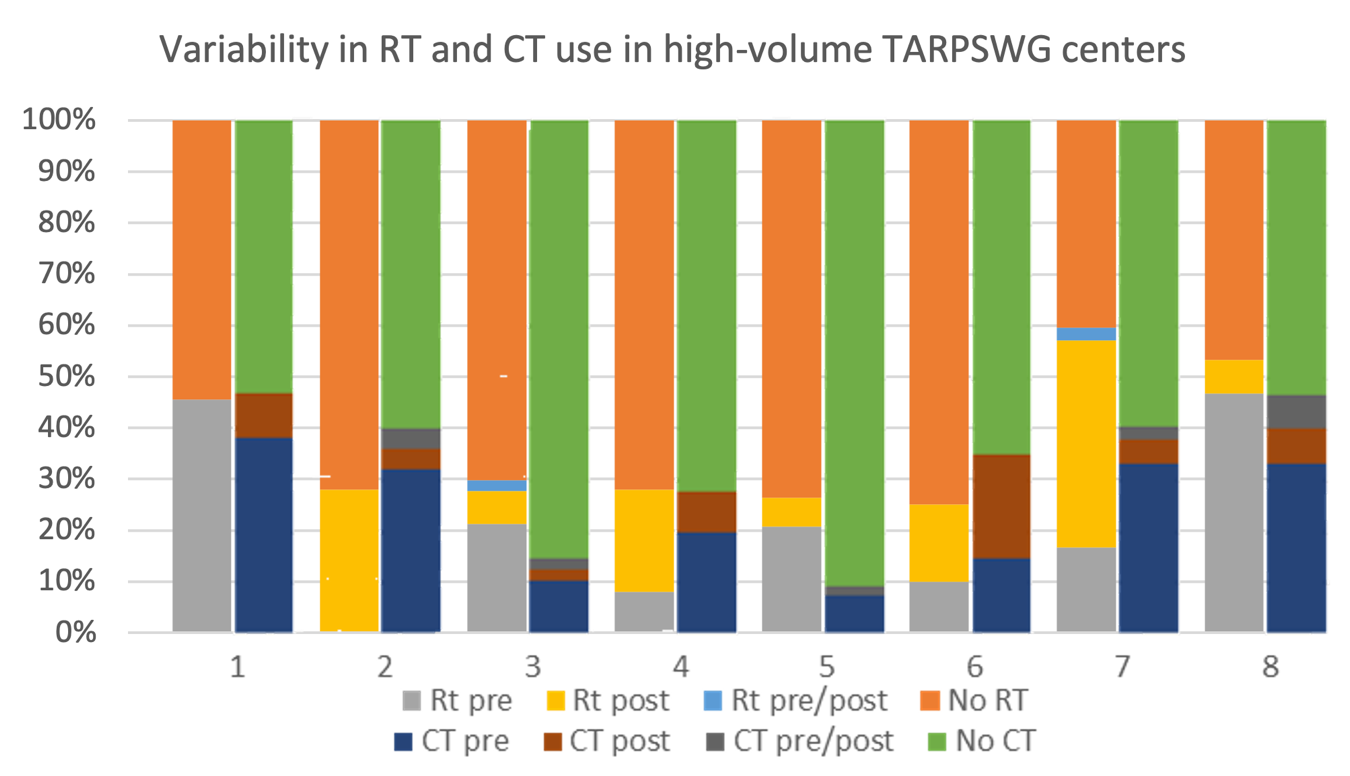
**

| **Table S2. Treatment modalities in non-operated patients according to histology** | | | | | | | |
| --- | --- | --- | --- | --- | --- | --- | --- |
|  | **Non-surgical patients n = 47** | **WDLPS**  **n= 1** | **DDLPS**  **n= 11** | **LMS n= 12** | **SFT**  **n= 4** | **Others**  **n=19** | **P** |
| **Sex, n (%)** | | | | | | | 0.66 |
| **Female** | 19 (40.4) | 0 | 4 (8.5) | 7 (14.9) | 1 (2.1) | 7 (14.9) |  |
| **Male** | 28 (59.6) | 1 (2.1) | 7 (14.9) | 5 (10.6) | 3 (6.4) | 12 (25.5) |  |
| **Age,** median (IQR) | 61 (47-76) | 69 | 61  (54-79) | 62  (47-77) | 60  (42-77) | 54  (43-75) | 0.850 |
| **ECOG, n(%)** | | | | | | | 0.74 |
| **0** | 15 (33.3) | 0 | 3 (6.7) | 5 (11.1) | 1 (2.2) | 6 (13.3) |  |
| **1** | 12 (26.7) | 1 (2.2) | 3 (6.7) | 2 (4.4) | 2 (4.4) | 4 (8.9) |  |
| **2** | 12 (26.7) | 0 | 2 (4.4) | 5 (11.1) | 1 (2.2) | 4 (8.9) |  |
| **3** | 6 (13.3) | 0 | 2 (4.4) | 0 | 0 | 4 (8.9) |  |
| **Tumor size (mm)**  median (IQR) | 130  (83-150) | 145 | 140  (120-200) | 101 (57-135) | 104  (72-145) | 130  (96-165) | 0.168 |
| **Laterality** | | | | | | | 0.98 |
| **Left** | 16 (34) | 0 | 4 (8.5) | 4 (8.5) | 1 (2.1) | 7 (14.9) |  |
| **Right** | 14 (29.8) | 0 | 3 (6.4) | 5 (10.6) | 1 (2.1) | 5 (10.6) |  |
| **Central** | 17 (36.2) | 1 (2.1) | 4 (8.5) | 3 (6.4) | 2 (4.3) | 7 (14.9) |  |
| **FNCLCC Grade** | | | | | | | **0.02** |
| **G1** | 16 (34) | 1 (2.6) | 0 | 4 (10.5) | 3 (7.9) | 8 (21.1) |  |
| **G2** | 16 (34) | 0 | 7 (18.4) | 5 (13.2) | 1 (2.6) | 3 (7.9) |  |
| **G3** | 6 (12.8) | 0 | 0 | 2 (5.3) | 0 | 4 (10.5) |  |
| **Treatment** | | | | | | | 0.30 |
| **Wait and see** | 8 (17) | 0 | 2 (4.3) | 2 (4.3) | 3 (6.4) | 1 (2.1) |  |
| **CT** | 13 (27.7) | 1 (2.1) | 2 (4.3) | 5 (10.6) | 0 | 5 (10.6) |  |
| **CT + RT** | 6 (12.8) | 0 | 2 (4.3) | 1 (2.1) | 0 | 3 (6.4) |  |
| **RT** | 14 (29.8) | 0 | 4 (8.5) | 4 (8.5) | 1 (2.1) | 5 (10.6) |  |

BSC: best supportive care
